# Quantifying right ventricular diffuse fibrosis in tetralogy of Fallot - a novel customised approach for the challenges of the right ventricle

**DOI:** 10.1186/1532-429X-18-S1-O26

**Published:** 2016-01-27

**Authors:** Ee Ling Heng, Peter Kellman, Michael A Gatzoulis, James Moon, Peter Gatehouse, Sonya V Babu-Narayan

**Affiliations:** 1 grid.421662.50000000092165443NIHR Cardiovascular Biomedical Research Unit, Royal Brompton & Harefield NHS Foundation Trust and Imperial College London, London, UK; 2 grid.439338.6Department of Adult Congenital Heart Disease, Royal Brompton Hospital, London, UK; 3 grid.279885.90000000122934638National Heart, Lung and Blood Institute, National Institutes of Health, Bethesda, MD USA; 4 grid.439632.9Heart Hospital Imaging Centre, The Heart Hospital, UCLH & UCL, London, UK; 5 grid.439338.6Cardiovascular Magnetic Resonance Unit, Royal Brompton Hospital, London, UK

## Background

There are clear clinical drivers for right ventricular (RV) T1 mapping in patients with repaired tetralogy of Fallot (rTOF), in whom myocardial fibrosis is implicated in adverse clinical outcomes. However, considerable technical challenges exist, due to thin mobile RV wall with adjacent strong signals from blood and epicardial fat. We prospectively aimed to explore the possible clinical significance of RV diffuse fibrosis in rTOF compared to health.

## Methods

### Technical

**Figure 1:**
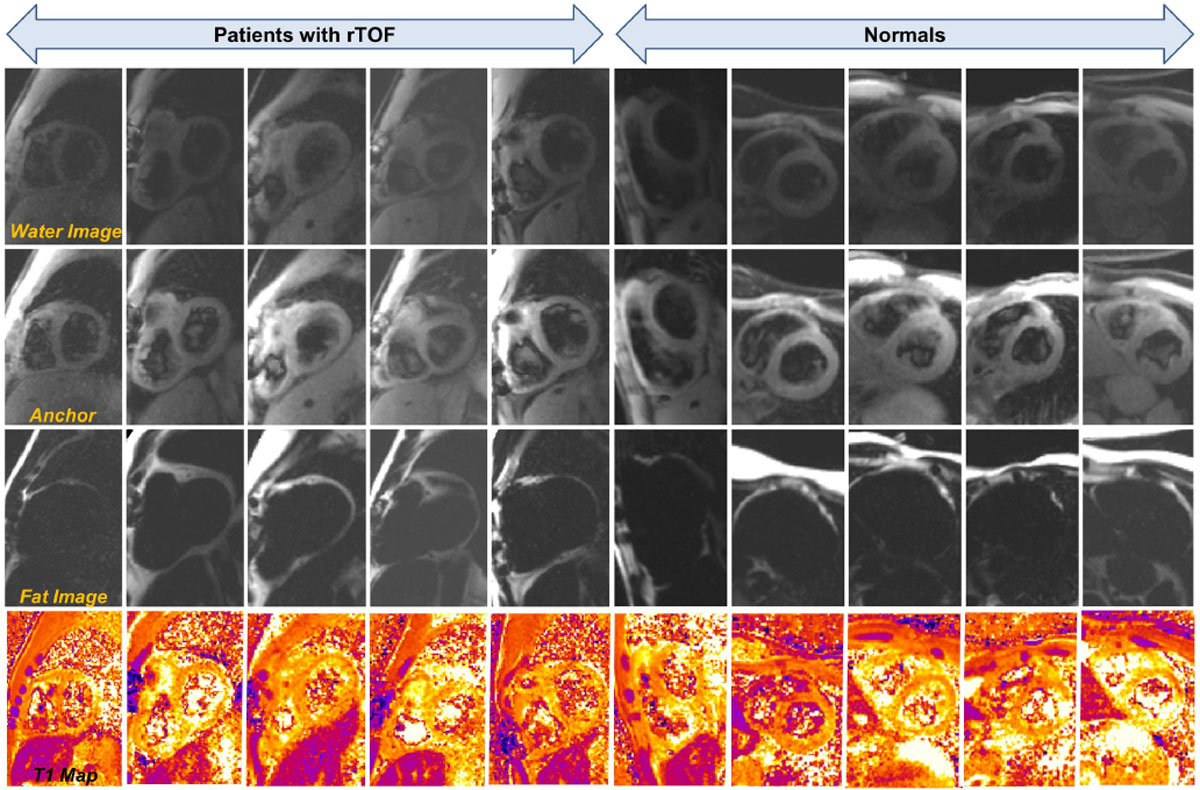
**Representative short-axis images from subjects scanned for RV T1 mapping by fat-water separated, MSPrep dark blood imaging**. Images for five subjects with repaired tetralogy of Fallot and five healthy volunteers shown (one subject per column): *Top row -* MoCo averaged water-only image at Ts 600 ms, *Second row -* MoCo averaged water-only anchor image at same window/level, *Third row -* MoCo averaged fat only image, *Bottom row -* T1 map generated from registration and 2-parameter fit of the six images per sampling scheme.

We adopted a saturation recovery (SASHA) based approach with MoCo averaging of single-shot fat-water separated images at 1.5T (Siemens Avanto, 32-channel thoracic phased-array).^4^ We applied Motion-sensitive dephasing (MSPrep) to null blood signal before each shot.^1-3^ Composite saturation and MSPrep parameters were optimised prior to four 10-cycle scans at saturation-recovery delay Ts≈600 ms and two "anchor" scans at Ts>6 sec,^5^ run free-breathing twice pre-contrast administration (Figure 1). Complex non-rigid MoCo averaging^6^ (fixed 50% acceptance) incorporated 5 shots at the most similar respiratory phase. Imaging parameters: TE = 1.0,2.7,4.3 ms (reduced to tolerate proximity to sternal wires), FA 10°, 6 × 1.9 × 2.1 mm acquired voxels, TGRAPPA rate 4, diastolic shot duration 175 ms. Post-contrast (0.15 mmol/kg Gadovist) RV T1 scans used Ts≈200 ms and "anchor" Ts>3 sec. Separate 11-HB MOLLIs were acquired for blood T1 and LV ECV, at a LV-aligned short-axis plane pre-contrast (5(3)3) and 15 minutes post-contrast (4(1)3(1)2).^7^

### Clinical

Data for 22 subjects (11 patients with rTOF and 11 age and gender-matched healthy volunteers) were acquired. Two-parameter fit pixelwise T1 maps were generated for each run. Mean RV free wall T1s were measured by two independent observers using CMR42. Inter-observer reproducibility was calculated by coefficient of variation (CoV%) = (within-subject standard deviation/mean) x100%.

## Results

**Figure 2:**
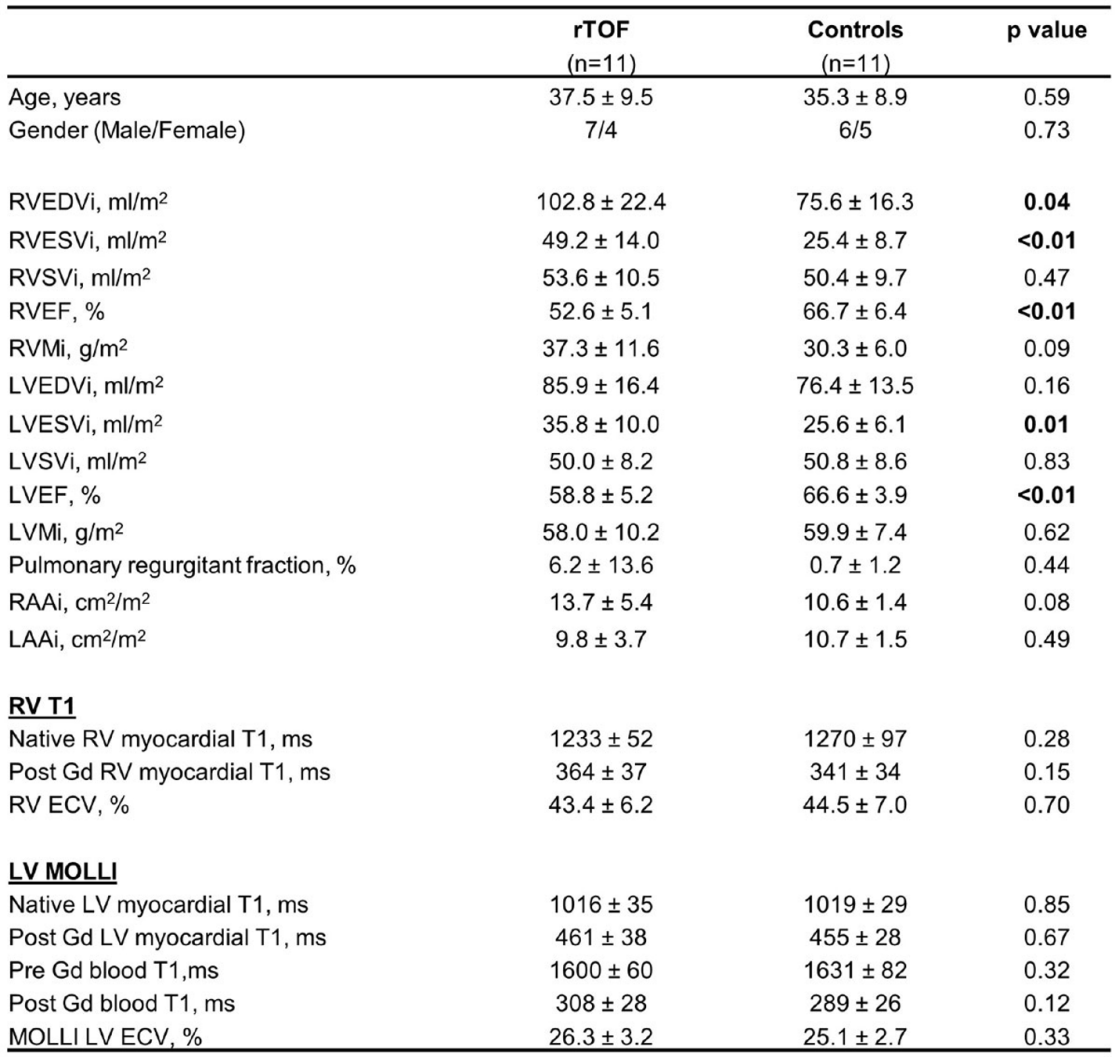
**Study subject demographics and T1 mapping results reported as mean ± standard deviation (SD)**. Note that MOLLI was used for LV values as the new method was targeted on the RV wall. **ECV**: extracellular volume fraction, **EDVi**: indexed end-diastolic volume, **ESVi**: indexed end-systolic volume, **SVi**: indexed stroke volume, **EF**: ejection fraction, **LAAi**: indexed left atrial area, **LV**: left ventricle, **Mi**: indexed mass, **RAAi**: indexed right atrial area, **rTOF**: repaired tetralogy of Fallot, **RV**: right ventricle

RV T1 maps were obtained in all subjects, with inter-observer reproducibility of native RV myocardial T1 (CoV 1.8%) and RV ECV (CoV 6.8%). There was no significant difference in RV native T1 and ECV of patients with rTOF compared to the controls who had thin RV wallls (Figure 2). This may reflect the modest sample size, or the inclusion of clinically stable patients with rTOF with minimal residual haemodynamic lesions, or may also reflect technical limitations. Saturation was optimised to <1% in the RV but non-uniformity over the heart requires investigation. The MSPrep required subject-specific optimisation for minimal partial volume contamination by blood which may explain high RV ECV. Known underestimation of native T1 by MOLLI compared to SASHA may also explain some of the RV-LV difference.

## Conclusions

RV T1 and ECV quantification is possible with the proposed technique but requires further development. The diagnostic value in this patient population merits further work towards a larger study and to explore histological correlation.

